# Copper Ions and Parkinson’s Disease: Why Is Homeostasis So Relevant?

**DOI:** 10.3390/biom10020195

**Published:** 2020-01-29

**Authors:** Marco Bisaglia, Luigi Bubacco

**Affiliations:** Molecular Physiology and Biophysics Unit, Department of Biology, Università di Padova, 35131 Padova, Italy

**Keywords:** copper, dopamine, Parkinson’s disease, α-synuclein, SOD1

## Abstract

The involvement of copper in numerous physiological processes makes this metal ion essential for human life. Alterations in copper homeostasis might have deleterious consequences, and several neurodegenerative disorders, including Parkinson’s disease (PD), have been associated with impaired copper levels. In the present review, we describe the molecular mechanisms through which copper can exert its toxicity, by considering how it can interfere with other cellular processes known to play a role in PD, such as dopamine metabolism, oxidative stress, and α-synuclein aggregation. The recent experimental evidence that associates copper deficiency and the formation of superoxide dismutase 1 (SOD1) aggregates with the progression of PD is also discussed together with its therapeutic implication. Overall, the recent discoveries described in this review show how either copper deficiency or excessive levels can promote detrimental effects, highlighting the importance of preserving copper homeostasis and opening unexplored therapeutic avenues in the definition of novel disease-modifying drugs.

## 1. Introduction

**Figure 1:**
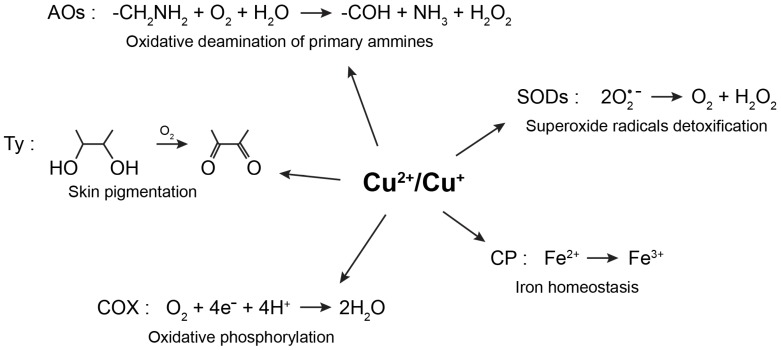
Physiological processes involving the redox activity of copper. Copper is often found as a cofactor in the active sites of enzymes with oxidoreductase activities. AOs—amine oxidases; COX—cytochrome c oxidase; CP—ceruloplasmin; Ty—tyrosinase; SODs—superoxide dismutases.

Copper is the third most abundant essential transition metal in humans, where the highest concentrations are found in the liver and brain [1,2]. Copper participates in several physiological processes, among others, skin pigmentation, preservation of the integrity of blood vessels, myelination, iron homeostasis, antioxidative defense, and neurotransmitter synthesis [3,4,5]. The essentiality of copper in a variety of biological processes is due to its role as a cofactor or structural component in numerous cuproproteins. More specifically, its oxidative state can change from Cu(I) to Cu(II) and the potential of this process can be finely tuned by the protein scaffold that binds the metal ion. Coherently, copper is often found as a cofactor in the active sites of enzymes with oxidoreductase activities (Figure 1). For example, Cu/Zn-superoxide dismutases catalyze the dismutation of superoxide radicals to oxygen and hydrogen peroxide, copper-containing amine oxidases catalyze the oxidative deamination of primary amines to aldehydes, releasing hydrogen peroxide and ammonia, and tyrosinase oxidizes biological phenols, such as tyrosine and dopamine, participating in this way to the formation of melanin pigments [4,5]. Mitochondrial cytochrome c oxidase, also known as Complex IV, is another extremely important copper-containing enzymatic complex, which receives electrons from cytochrome c molecules, and uses them to transform molecular oxygen into water [4,5]. Through its interaction with ceruloplasmin, copper takes also part in iron homeostasis. In fact, ceruloplasmin exhibits a copper-dependent oxidase activity, which transforms Fe(II) in Fe(III), assisting in such a way the transferrin-mediated transport of iron in the plasma [6]. In addition to being a cofactor in numerous redox enzymes, through its interaction with synaptic proteins and neurotransmitters receptors, copper has been demonstrated to play a key role at synapses [7]. Coherently, by modulating synaptic activity, excitotoxic cell death and signaling cascades induced by neurotrophic factors, copper participates in several neural cell functions [7]. Considering the numerous cellular pathways that require the presence of copper, it is not surprising that failures in the homeostatic processes leading to either copper excess or deficit can lead to severe disorders.

In the present review, the involvement of copper in Parkinson’s disease (PD) will be considered in light of the most recent discoveries. More specifically, a general picture is now emerging in which either excessive levels of copper or its deficiency appear both detrimental for PD, emphasizing the importance of a precise homeostatic control. Interestingly, the cellular pathways associated with both high and low copper levels lead to increased oxidative stress conditions, suggesting that converging intracellular stressful conditions could mediate cell damage in both scenarios.

## 2. Copper Metabolism

**Figure 2:**
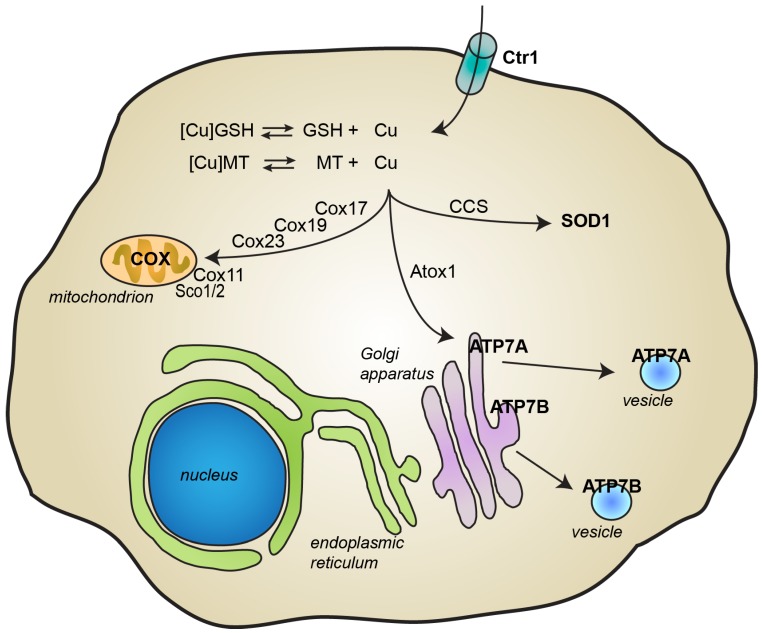
Cellular metabolism of copper. Copper import inside cells is mainly mediated by the copper transporter Ctr1. Inside cells, glutathione (GSH) and metallothioneins (MT) contribute to maintaining levels of free copper very low. Copper is transported to its final destination through the action of several specific chaperones. Copper transport protein Antioxidant-1 (Atox1) delivers copper to the P-type ATPases ATP7B and ATP7B, which are then responsible for the incorporation of copper into newly synthesized cuproproteins and for copper excretion through the plasma membrane. Copper chaperone for SOD1 (CCS) delivers copper to superoxide dismutase 1 (SOD1). At least 6 chaperones (Cox 11, Cox17, Cox19 and Cox23, and Sco1 and Sco2) deliver copper inside mitochondria into cytochrome c oxidase.

Copper is essentially acquired by humans through the diet and it is then delivered to organs, cells, and proteins that require its presence to exert their physiological function. Ceruloplasmin is the main protein involved in copper transport inside the body, being able to bind six copper ions per protein [5]. Other proteins in the plasma, such as albumin, also participate in copper transport [5]. As represented in Figure 2, copper import inside cells is mainly dependent on the copper transporter Ctr1 [3,8,9,10]. Under physiological conditions where copper is mostly cuprous Cu(I), the concerted action of metallothioneins and other low molecular weight ligands, such as glutathione, contribute to keeping the concentration of free copper at extremely low levels (10^−18^ M) [8,11]. Cytosolic copper can be then transferred to its final destinations by the action of numerous and specific chaperones. At least six proteins, the soluble proteins Cox17, Cox 19, and Cox 23, and the mitochondrial inner membrane proteins Sco1, Sco2, and Cox11, have been classified as copper chaperones for the mitochondrial cytochrome c oxidase complex and are required to both transport copper from the cytosol to mitochondria and to incorporate the metal ion into two different subunits of complex IV [3,8,9,12]. The copper chaperone for superoxide dismutase 1 (SOD1) (CCS) is responsible for the maturation of SOD1, which eventually leads to SOD1 activation, while the copper chaperone Atox1 delivers copper to the P-type copper-transporting ATPases ATP7A and ATP7B [3,8,9,12]. These proteins are typically located in the trans-Golgi network, where they are responsible for loading copper into cuproenzymes, including dopamine-β-hydroxylase, lysyl oxidase, tyrosinase, and ceruloplasmin [8]. ATP7A and ATP7B are also involved in copper secretion from cells [3,8,9]. For instance, when copper levels increase, ATP7A translocates to the plasma membrane to facilitate copper export. ATP7A protein is also responsible for the transport of copper through the basolateral membrane of intestinal epithelial cells into the circulation [3]. ATP7A seems to play a crucial role also in copper release at synapses [7]. In contrast, by delivering copper from the liver into the bile, ATP7B contributes to the elimination of excessive levels of copper from the body [3].

## 3. Alteration of Copper Homeostasis and Pathological Consequences

In light of its essential role for life, alterations in copper homeostasis, which may lead to either higher or lower levels, can have deleterious consequences. Acute toxicity induced by copper intake is a quite rare event as the amount of copper required to induce adverse effects rather high, in the order of grams. The principal targets of acute copper-related toxicity are gastrointestinal, hepatic, renal, hematological, and cardiovascular systems and the symptoms include abdominal pain, nausea, vomiting, hypotension, tachycardia, intravascular hemolysis, liver, and renal failure [13]. In contrast to acute intoxication due to high copper levels, chronic copper dyshomeostasis is often the result of genetically determined disorders, such as Menkes disease and Wilson disease [3]. Menkes disease is an X-linked recessive disorder, caused by mutations in the *ATP7A* gene coding for the copper-transporting ATPase ATP7A. It is a fatal metabolic disease, characterized by progressive neurological degeneration, connective tissue abnormalities, muscular hypotonia, hypothermia, and abnormalities of the skin and hair [14,15]. Due to the requirement of ATP7A for the transport of copper through the basolateral membrane of intestinal epithelial cells, in Menkes disease, a copper deficiency is present in blood, kidney, liver and brain, while copper deposits are present in the intestinal enterocytes [14,15]. Wilson disease is an autosomal recessive disorder caused by mutations in the *ATP7B* gene coding for the copper-transporting ATPase ATP7B [3,5]. The impaired activity of ATP7B leads to copper accumulation in multiple organs, mainly in the liver, but also in the brain where the copper content was shown to be approximately eight times higher than controls [16]. Interestingly, it has been reported that Wilson disease patients manifest Parkinson-like symptoms, such as tremor, bradykinesia, and postural instability [17]. The role of copper in Menkes and Wilson diseases has been extensively reviewed elsewhere [13,14,17]. In addition to the aforementioned genetically determined disorders, aging-dependent alterations in copper levels seem to contribute to the onset of neurodegenerative disorders, including Alzheimer’s disease, Huntington disease, and Parkinson’s disease [3,8].

## 4. Parkinson’s Disease

PD is a debilitating aging-related neurodegenerative disorder, which mainly affects the locomotion capability of patients. Although nonmotor symptoms could appear during the disease progression, the principal clinical features are resting tremor, muscular rigidity, bradykinesia, and postural instability [18]. The two pathological hallmarks of the disease are the preferential loss of dopaminergic neurons in the substantia nigra and the presence of intracellular proteins aggregates, referred to as Lewy bodies, which are mainly composed of α-synuclein fibrils [18]. While approximately 10% of PD cases have a genetic origin, almost 90% are considered sporadic, suggesting that aging and environmental factors could play a crucial role in the onset of the disease. Nevertheless, the most accepted point of view is that genetic susceptibilities and environmental factors cooperate all together in the etiology of PD. Although the pathogenesis of PD has not been fully elucidated, a general consensus exists on the role exerted by oxidative stress and mitochondrial dysfunction in the disease progression. Post-mortem analyses on PD brains, when compared to controls, revealed increased levels of oxidative damages on proteins, DNA, and lipids [18]. Mitochondrial dysfunction, associated with impairment of complex I and III, was also observed [18]. Moreover, the role of oxidative stress and mitochondrial dysfunction in promoting nigrostriatal neurodegeneration has been substantiated by studies carried out on both sporadic (i.e., toxin-based) and genetic models of PD [18]. A 40%-90% reduction of glutathione levels in substantia nigra tissue from PD patients during the advancement of the disease has also been reported with the concomitant increase of iron levels [19,20,21,22].

The role of heavy metals, among which copper, in promoting the onset of PD is supported by several epidemiological studies. For instance, occupational long-term exposure to copper, iron, manganese, lead, etc., alone or in combination, has been associated with an enhanced risk of developing PD [23,24,25,26,27]. Interestingly, as recently reviewed, synergistic effects may derive from the combination of different metals [28]. This is the case, for instance, of the combined exposure to iron/copper, lead/copper, lead/iron, mercury/manganese, etc. when compared with the effects of single metals [28].

## 5. Mechanisms of Copper Toxicity in Parkinson’s Disease

The molecular mechanisms through which copper dyshomeostasis can promote the onset of PD are not fully understood and controversial hypotheses have been suggested as described below. A first mechanism relies on the ability of free copper to bind to cysteine residues of proteins, with the consequence that such an interaction can result in the inactivation of their enzymatic activity [29]. For instance, the pretreatment of rat striatal homogenates with metal cations having significant reactivity toward thiols (Cd^2+^, Cu^2+^, Hg^2+^), as well as with the -SH alkylating agent N-ethylmaleimide (NEM), have been described to decrease the specific binding sites in D2 dopamine receptors as measured through a standard [^3^H]-spiperone binding assay [30]. More precisely, the administration of 3 mM copper resulted in a 40%-60% reduction in the binding of D2 dopamine receptors with [^3^H]-spiperone [30], confirming that thiol modifications induced by copper can have functional consequences.

### 5.1. Copper and Oxidative Stress

**Figure 3:**
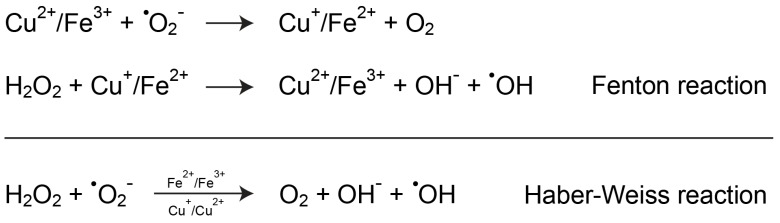
Copper-mediated increase of oxidative stress. Copper can participate in Fenton and Haber–Weiss reactions, which lead to the formation of the highly reactive hydroxyl radical intermediate, either directly or indirectly by interfering with iron homeostasis.

Besides its direct interaction with the sulfhydryl groups of proteins, copper toxicity mainly relies on its redox activity, the same property that is exploited by numerous enzymes and that makes copper biologically essential. Accordingly, a widely accepted mechanism through which copper could participate in the pathogenesis of PD is its ability to increase oxidative stress by catalyzing noxious redox reactions that involve oxygen derivatives. As represented in Figure 3, copper, like iron, could participate in the conversion of the superoxide anion and hydrogen peroxide into the hydroxyl radical through the Fenton and Haber–Weiss reactions. This latter species has a very short half-life in the range of 10^−9^ s and is considered the most reactive oxygen species, being able to interact with practically any type of biomolecule at a diffusion-controlled rate [18].

### 5.2. Copper and Dopamine Oxidation

As aforementioned, PD is characterized by the preferential degeneration of dopaminergic neurons and growing evidence points out on a possible role of dopamine itself in promoting cell death. In contrast to neurons containing other neurotransmitters, dopamine can make dopaminergic neurons particularly susceptible to oxidative damage. After its synthesis, dopamine is almost completely sequestered inside synaptic vesicles, where it can reach concentrations up to 1 M [31] and where it is stabilized by the low pH value of vesicle lumen. However, the cytosolic fraction of dopamine can undergo a spontaneous autoxidation process, which leads to the formation of both reactive oxygen species and dopamine quinones (DAQs) [32]. This latter pathway is confirmed by the presence of neuromelanin, a dark polymer, which is formed by the polymerization of DAQs and DAQ-modified proteins and which also incorporate both lipids and metal ions [33]. Due to its ability to sequester both reactive dopamine-quinones and redox-active metal ions, neuromelanin is considered to provide a protective mechanism that prevents neurotoxicity [33]. Copper has been demonstrated to increase the oxidation process of dopamine leading to a variety of potentially toxic species, such as dopamine-quinones, ·O_2_^−^, H_2_O_2_, and hydroxyl radical [33,34,35]. Interestingly, the locus coeruleus and substantia nigra, where neuromelanin is mostly found, are the brain regions with the highest levels of copper [36,37,38]. The presence of copper inside neuromelanin suggests its active participation in dopamine oxidative polymerization [33], even though, as an alternative hypothesis the presence of such a unique metal-binding pigment in the human substantia nigra and locus coeruleus could account for the high levels of copper in these brain regions [9]. The presence of labile pools of copper ions in the brain, as demonstrated by Dodani SC and co-workers [39], is probably the major source of dopamine oxidation. In addition to the direct reactivity of copper towards dopamine, the oxidation can also be mediated by copper ions bound to coordinating ligands or peptides and proteins involved in neurodegenerative processes, as recently reviewed [33]. In the context of PD, a major role in promoting copper-induced dopamine oxidation could be played by the protein α-synuclein. The production of reactive oxygen species, mediated by α-synuclein oligomers, which could be inhibited by the presence of copper chelating agents [40], supports such a possibility even though direct in vivo evidence is still lacking.

### 5.3. Copper Effects on α-Synuclein Aggregation

The protein α-synuclein is unanimously recognized as a central player in the pathogenesis of PD. First, as aforementioned, the fibrillar form of the protein represents the major component of Lewy bodies, a pathological hallmark of PD. Moreover, autosomal dominant mutations in the gene coding for the protein, as well as the duplication and triplication of the gene, have been associated with familial forms of PD [41]. α-Synuclein is a natively unfolded protein, able to interact with membranes adopting an α-helical conformation [42]. Under pathological conditions, the protein aggregates in oligomers and fibrils forming toxic amyloidogenic conformations, particularly rich in β-sheet structures [42]. Earlier in vitro analyses indicated that the presence of millimolar concentrations of various metal ions, among which copper, promotes the formation of partially folded amyloidogenic conformations that are more prone to aggregate [43,44,45]. Although it is worth mentioning that millimolar metal concentrations do not necessarily provide an accurate picture of the in vivo situation, the pathophysiological relevance of the aforementioned discoveries arises from the fact that both α-synuclein and metals are found at synapses, where they participate in synaptic functions [7,46]. Moreover, the contribution of the single metals can result in synergistic effects on α-synuclein aggregation [28,46]. The interaction between α-synuclein and metal ions has been proposed to stabilize a partially folded conformation of the protein by decreasing the electrostatic repulsion between the negative charges in this protein, which are mostly present in its C-terminal region, between residues 116-127 [47,48]. In addition to the C-terminus binding site, another copper-binding site with a nanomolar affinity has been described in the N-terminal region of the protein [47,49,50,51,52,53]. The formation of a complex with copper at the N-terminus seems to involve the amino-terminal group of Met1, backbone amide nitrogen, and carboxylate of Asp2 and imidazole group of His50, even though a general consensus does not exist [54]. Interestingly, copper has been described to accelerate α-synuclein fibril formation even at physiologically relevant concentrations without altering fibrils morphology [44,49]. While most of the studies carried out to evaluate the effects of copper on α-synuclein were done in the presence of Cu(II), less information has been produced with Cu(I). The experimental data available suggest that, also in the case of Cu(I), the protein possesses two binding sites located at the N- and C-termini, with a comparable affinity in the micromolar range [51,55]. The first binding site involves the sulfur atoms of Met1 and Met5 thioether groups, whereas in the other one the thioether groups from Met116 and Met127 are able to bind the cuprous ion [51,55].

While the global picture that emerges from the aforementioned studies supports a direct role of copper in promoting α-synuclein aggregation, some caution must be used when considering the pathological relevance of the copper/α-synuclein interaction. In fact, recent evidence revealed that Lewy bodies, extracted from brain tissues, contain α-synuclein acetylated at the N-terminal group and the N-acetylated form of the protein is commonly found in vivo [56]. The acetylated form of the protein has increased helical folding propensity, membrane binding affinity, and resistance to aggregation [57,58,59,60]. Even though the protein still retains its capability to bind Cu(I) [61], the principal consequence of N-acetylation is that the capability of α-synuclein to bind Cu(II) is strongly affected [54,62]. The physiopathological implication of the interaction between α-synuclein and copper has been extensively described elsewhere [9,54,63,64,65].

## 6. Copper Deficiency in Parkinson’s Disease

While most, if not all the aforementioned studies associate increased copper levels with increased PD risk, this correlation is still controversial and far from being definitely elucidated. In fact, recent studies support the hypothesis that reduced copper levels are correlated to an enhanced risk of developing the disease. More specifically, in PD patients the blood concentration of copper, ceruloplasmin, and its oxidase activity, as well as the copper atoms per ceruloplasmin molecule were lower in comparison to age-matched healthy individuals [66,67]. Moreover, copper levels have been demonstrated to be lower also in the most affected brain regions of PD patients in comparison to age-matched control individuals, with a 35%-50% reduction of copper content of the substantia nigra and locus coeruleus [68,69,70,71,72]. To confirm that reduced copper levels were not the result of the marked degeneration of the copper-rich neuronal populations in these brain regions, Davies and co-workers analyzed intraneuronal copper at the single-cell level through synchrotron radiation X-ray fluorescence microscopy and particle-induced X-ray emission microscopy, showing a 55%-65% reduction in copper levels in both the substantia nigra and locus coeruleus from PD brains [72].

Through its binding to ceruloplasmin, copper stimulates its ferroxidase activity and participates in iron homeostasis, so that some indirect toxicity mediated by altered concentrations of iron could be a consequence of low levels of copper. Accordingly, aceruloplasminemia, an autosomal recessive deficiency of ceruloplasmin caused by mutations in the *ceruloplasmin* gene, is associated with the accumulation of iron in the liver, pancreas, retina, and basal ganglia [73,74,75,76]. Moreover, iron deposition in the brain has been described to be associated with neuronal loss in the same regions and the effects seem to be related to the capability of the ferrous ion to increase oxidative conditions through the Fenton and Haber–Weiss reactions [73,74]. Interestingly, among the neurological symptoms of aceruloplasminemia, loss of motor coordination and other motor deficits overlap with some clinical manifestations associated with PD.

## 7. A New Proposed Role of SOD1 in Parkinson’s Disease Pathology

Recent work has associated the formation of SOD1 amorphous aggregates with the progression of PD, further linking copper deficiency to the disease [77]. In fact, the investigators have detected SOD1 immunoreactivity in Lewy bodies and Lewy neurites in both the substantia nigra and locus coeruleus of PD brains, confirming previous reports of co-deposition of α-synuclein and SOD1 in PD-associated Lewy pathology [78]. However, in addition to these amyloid aggregates, morphologically distinct SOD1-containing aggregates were also observed in the same brain regions. These new SOD1-containing aggregates, which presented amorphous and spherical structures, were described to be practically devoid of α-synuclein, while containing ubiquitin, suggesting an impairment in their proteasomal degradation pathway [77]. Even more interestingly, not only the density of SOD1 amorphous aggregates was significantly higher in locus coeruleus and substantia nigra of PD brains in comparison to age-matched controls, but it was also significantly more elevated than all nondegenerating regions of the same PD brains, suggesting an association between SOD1 aggregation and neuronal degeneration [77]. The link with copper arose from the observation of a positive shift in the isoelectric point of soluble SOD1 extracted from PD brains in comparison to controls, a behavior that was previously demonstrated to be dependent on a decreased incorporation of the copper metal ion into the protein [79]. Through the use of specific antibodies directed towards metal-deficient misfolded SOD1, the investigators confirmed their presence in the vulnerable regions of PD brains, while they were absent in the same regions of control brains [77]. As SOD1-specific activity was strongly impaired in copper-deficient substantia nigras in comparison to other nondegenerating brain regions, the investigators proposed a model in which copper deficiency in the substantia nigra and locus coeruleus is associated with a reduction in copper-loaded SOD1 with the consequence that the less stable apo-protein accumulates within amorphous aggregates and loses its ability to protect neurons from oxidative damage [77].

## 8. A therapeutic Conundrum

Whether copper toxicity in PD is due to increased levels or to a deficiency of this metal ion (or both through different mechanisms), the therapeutic approaches have to be very different.

A possible strategy to cope with the damage induced by copper accumulation is the use of specific chelators able to cross the blood-brain barrier. A similar strategy has been exploited to deal with iron toxicity in animal models of PD, as reviewed elsewhere [80,81]. In light of the positive outcomes observed, two independent, small scale, clinical trials have been carried out to test the protective effects of the iron chelator deferiprone in PD patients with positive results in both cases [82,83]. In contrast to iron, the results obtained with copper chelators were disappointing. Not only the use of the relatively specific copper chelator D-penicillamine did not show any protection in an N-methyl-4-phenyl-1,2,3,6-tetrahydropyridine (MPTP)-based mouse model of PD [84], but in the presence of diethyldithiocarbamate, another effective copper chelator, enhanced neurotoxicity was observed in the same toxin-based experimental model [85]. However, it has to be mentioned that the MPTP-based model may lead to neurodegeneration through a copper-independent mechanism. Recently, more positive results have been described. The use of 8-hydroxyquinoline-2-carboxaldehyde isonicotinoyl hydrazone (INHHQ), a moderate metal-binding compound, was able to efficiently compete with α-synuclein for both Cu(I) and Cu(II) binding, fully inhibiting the protein aggregation process both in vitro and in cells [86]. Moreover, INHHQ has been demonstrated to be safe when injected in mice and to be able to cross the blood-brain barrier [86]. Considering these promising results, further studies are required to evaluate the effects of INHHQ in more physiopathologically relevant models of PD.

The experimental evidence supporting the hypothesis that low copper levels are associated with PD implies that the use of molecules able to deliver copper into the brain might have beneficial effects on the progression of PD. A similar approach, with the use of the copper-containing compound diacetylbis(N(4)-methylthiosemicarbazonato) copper(II) (Cu^II^-ATSM), has been already described as a valid therapeutic strategy towards amyotrophic lateral sclerosis (ALS). In fact, the protective effects of Cu^II^-ATSM have been demonstrated in several mouse models of ALS where the molecule has also been shown to restore the functionality of copper-deficient SOD1 [87,88,89]. The very positive readouts led to the clinical evaluation of Cu^II^-ATSM, which is now used in one phase 2/3 clinical trial (NCT04082832).

Cu^II^-ATSM is an orally bioavailable, blood-brain barrier-permeable molecule that has traditionally been used in cellular imaging experiments to selectively label hypoxic tissues [90]. It is also able to accumulate in cells with over-reduced states associated with mitochondrial dysfunction [91] and, interestingly, it has been demonstrated to accumulate within the striatum of PD patients with an accumulation level that positively correlates with disease stage [92]. It is also worthy to note the fact that, in a previous work based on both sporadic (toxin-based) and genetic mouse models of PD, Cu^II^-ATSM has been demonstrated to rescue dopaminergic cell loss and improve motor dysfunction [93]. These positive results, together with the ability of Cu^II^-ATSM to accumulate in PD-relevant tissues, with the aforementioned purported role of SOD1 in the pathogenesis of PD and with the fact the Cu^II^-ATSM is able to stimulate SOD1 activity, represent the rationale for a phase 1 clinical trial based on the use of Cu^II^-ATSM, which is currently in progress (NCT03204929).

## 9. Conclusions

**Figure 4:**
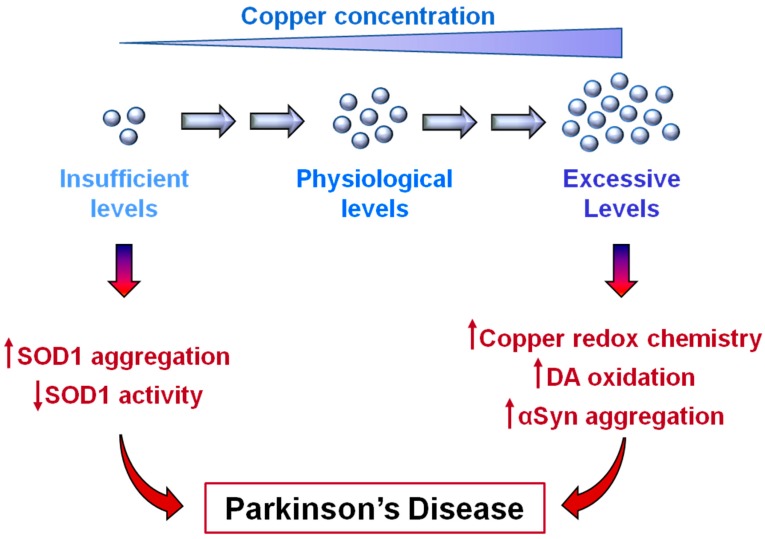
Copper dishomeostasis in Parkinson’s disease (PD). Copper deficiency, as well as excessive copper levels, can promote cellular events associated with PD. Low levels of copper are responsible for SOD1 aggregation while hindering SOD1 maturation. High levels of copper promote α-synuclein aggregation and increase oxidative conditions through Fenton and Haber–Weiss reactions and by favoring dopamine (DA) oxidation.

PD is still an incurable disorder and the current therapeutic treatments are directed at improving clinical symptoms without affecting the progression of the disease. One of the reasons why an efficacious therapy to fight the disease progression is still lacking is probably related to the multifactorial nature of PD, in which genetic susceptibilities and environmental factors contribute to the onset of the disorders. The consequence is that the molecular and cellular pathways affected in PD have not been fully elucidated and the global knowledge of the pathogenesis of the disease is still elusive. While the epidemiological correlation between the chronic exposure to heavy metals, among which copper, and a higher risk of developing PD has been recognized for a long time now, the role of copper dyshomeostasis in PD has been explored only more recently. As described in the previous sections, copper is found as a cofactor in the active sites of several enzymes, so that it takes part in a number of enzymatic reactions inside cells. The picture that is now emerging is that the redox activity that makes copper essential for human life can be harmful in the presence of excessive levels of this metal ion. However, copper deficiency also appears to induce detrimental consequences due to the loss of its biological functions. Interestingly, most of the molecular processes that correlate excessive copper levels with neurotoxicity converge on the enhancement of intracellular oxidative conditions. Similarly, by interfering with the SOD1 maturation process, copper deficiency is also associated with increased oxidative damage, suggesting that analogous molecular mechanisms of toxicity could mediate cell damage in both copper dyshomeostatic situations. In conclusion, the recent discoveries described in this review, summarized in Figure 4, highlight the importance of preserving copper homeostasis, opening new therapeutic avenues in the definition of novel disease-modifying drugs. Coherently, a phase 1 clinical trial, based on the use of the copper-delivering molecule Cu^II^-ATSM, has been recently started and is currently under evaluation.
